# Nanopore Fabrication by Controlled Dielectric Breakdown

**DOI:** 10.1371/journal.pone.0092880

**Published:** 2014-03-21

**Authors:** Harold Kwok, Kyle Briggs, Vincent Tabard-Cossa

**Affiliations:** Department of Physics, University of Ottawa, Ottawa, Ontario, Canada; Wake Forest University School of Medicine, United States of America

## Abstract

Nanofabrication techniques for achieving dimensional control at the nanometer scale are generally equipment-intensive and time-consuming. The use of energetic beams of electrons or ions has placed the fabrication of nanopores in thin solid-state membranes within reach of some academic laboratories, yet these tools are not accessible to many researchers and are poorly suited for mass-production. Here we describe a fast and simple approach for fabricating a single nanopore down to 2-nm in size with sub-nm precision, directly in solution, by controlling dielectric breakdown at the nanoscale. The method relies on applying a voltage across an insulating membrane to generate a high electric field, while monitoring the induced leakage current. We show that nanopores fabricated by this method produce clear electrical signals from translocating DNA molecules. Considering the tremendous reduction in complexity and cost, we envision this fabrication strategy would not only benefit researchers from the physical and life sciences interested in gaining reliable access to solid-state nanopores, but may provide a path towards manufacturing of nanopore-based biotechnologies.

## Introduction

Nanopore sensing relies on the electrophoretically driven translocation of biomolecules through nanometer-scale holes embedded in thin insulating membranes to confine, detect and characterize the properties or the activity of individual biomolecules electrically, by monitoring transient changes in ionic current [1]–[4]. The field was initially shaped by the ability of researchers to exploit biological channels to translocate single molecules [5], [6]. It rapidly expanded when new techniques to fabricate individual molecular-sized holes in thin solid-state materials were developed over the last decade [7]–[12]. These techniques, based on beams of high-energy particles, either produced by a dedicated ion beam machine (i.e ion-beam sculpting) or a transmission electron microscope (i.e TEM drilling), allowed researchers to control the nanopore size at the sub-10-nm length scale with single nanometer precision, thus greatly diversifying the breadth of applications. Since then, a host of applications for DNA, RNA and proteins analysis using both biological and solid-state nanopores have been demonstrated [4], [13], [14]. Compared to their organic counterparts, solid-state nanopores were expected to emerge as an essential component of any practical nanopore-based instrumentation due to the size control, increased robustness of the membrane, and their natural propensity for integration with wafer-scale technologies, including CMOS and microfluidics [15], [16]. Yet, this prospect is significantly hindered due to the constraints and limitations imposed by ion beam sculpting and transmission electron microscopy-based drilling, which, to this date, remain the only viable tools for achieving nanopores fabrication with dimensional control at the 1-nm scale. The complexity, low-throughput, and high-cost associated with these techniques restrict accessibility of the field to many researchers, greatly limit the productivity of the community, and prevent mass production of nanopore-based devices. Alternative nanofabrication strategies are therefore needed for the field to continue to thrive, and for the promised health-related applications to be successfully commercialized (including single-molecule DNA sequencing). Here, we introduce a fabrication technique based on the use of high electric fields to control dielectric breakdown in solution. The method is automated, simple, and low-cost, allowing nanopores to be created directly in aqueous solution with sub-nm precision, greatly facilitating use and improving yield of functional solid-state nanopore devices. We envision this fabrication strategy will not only provide a path towards nanomanufacturing of nanopore-based devices for a wide range of biotechnology applications, but will democratize the use of solid-state nanopores, while offering researchers new strategies for designing nanofluidics devices, as well as integrating nanopores with CMOS and microfluidics technologies.

## Results and Discussion

**Figure 1:**
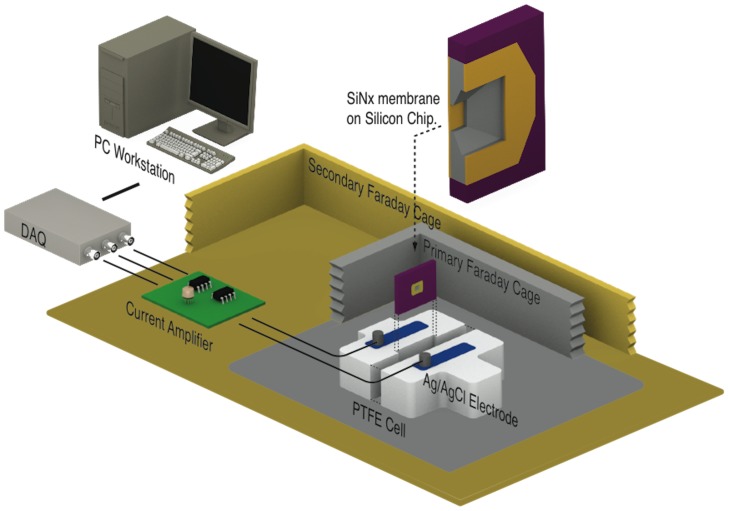
Schematic of the fabrication setup. A computer-controlled custom current amplifier is used to apply voltages up to ±20 V and measure the current with sub-nA sensitivity from one of the two Ag/AgCl electrodes positioned on either sides of the membrane. It is noteworthy to realize that this experimental setup is identical (with the exception of the custom current amplifier replacing the commonly used Axopatch 200B) to the instrumentation used to study DNA or proteins translocation through nanopores.

We fabricate individual nanopores on thin insulating solid-state membranes directly in solution. A thin silicon nitride (SiN_x_) membrane, supported by a silicon frame, is mounted in a liquid cell and separates two reservoirs containing an aqueous solution of 1M KCl. Ag/AgCl electrodes immersed on both sides of the membrane are connected to a custom-built resistive feedback current amplifier, which allow trans-membrane potentials of up to ±20 V to be applied. The setup shown in Figure 1 is otherwise identical to what is commonly used for biomolecular detection [17], which greatly facilitates the transition to sensing experiments, eliminating further handling of membranes. See Material and Methods section and Section S1 for more detail.

**Figure 2:**
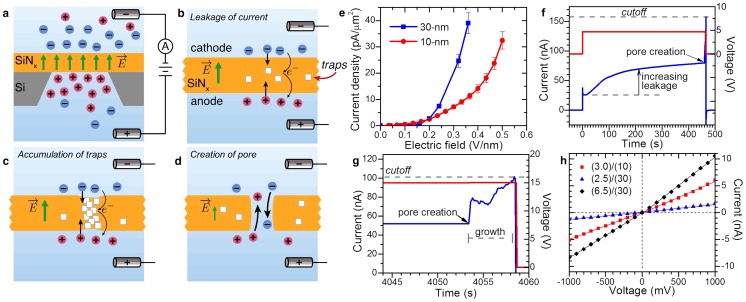
Nanopore formation by dielectric breakdown. a) Application of a trans-membrane potential generates an electric field inside the SiN_x_, and charges the interfaces with opposite ions. b) Leakage current through the membrane follows a trap-assisted tunneling mechanism. Free charges (electrons or holes) can be produced by redox reactions at the surface or by field ionization of incorporated ions. The number of available charged traps (structural defects) sets the magnitude of the observed leakage current. c) Accumulation of charge traps produced by electric field-induced bond breakage or energetic charges carries leads to a highly localized conductive path, and a discrete dielectric breakdown event. d) A nanopore is formed following removal of the defects. e) Leakage current density for SiN_x_ membranes (50-µm× 50-µm). The leakage current is fully reversible and stable, unless high fields are sustained, see Section S2 f) Leakage current at 5 V, on a 10-nm-thick SiN_x_ membrane, in 1 M KCl at pH13.5. Pore created is ∼5-nm (18 nS). The slowly increase leakage current, following the capacitive spike, is a result of the accumulation of traps in the membrane. g) Experiment performed at 15 V, on a 30-nm-thick SiN_x_ membrane, in 1 M KCl pH10. The nanopore is allowed to grow until a pre-determined threshold current is reached, at which point the voltage is turned off. The observed current fluctuations at the onset of pore formation are attributed to significant low-frequency noise at this voltage. Pore created is ∼3-nm (2.9 nS). h) Current-to-voltage curves for 3 nanopores fabricated on different membranes. The legend indicates the (pore diameter)/(membrane thickness) in nm. Measurements performed in 1 M KCl pH8, with an Axopatch 200B.

A single nanopore is fabricated by applying a constant potential difference, Δ*V*, across a *t*  = 10-nm or 30-nm thick SiN_x_ membrane, to produce an electric field, *E* = Δ*V*/*t* in the dielectric membrane in the range of 0.4-1 V/nm (Figure 2a). At these high field strengths, a sustainable leakage current, *I_leakage_*, is observed through the membrane, which remains otherwise insulating at low fields. *I_leakage_* rapidly increases with electric field strength, but is typically tens of nanoamperes for our operating conditions. We attribute the dominant conduction mechanism to a form of trap-assisted tunneling of electrons, supplied by ions in solution [18]–[21] (Figure 2b and 2c), since the membrane is too thick for significant direct tunnelling [18], and migration of impurities cannot produce lasting currents [22]. Direct migration of electrolyte ions is also unlikely, or negligible, since for a given electric field strength, a higher *I_leakage_* is observed in thicker membranes (Figure 2e). A larger current is observed on thicker membranes since the number of charge traps (defects) per unit area is greater, as their number in the material increases with volume. We provide additional discussion on the characteristics of the leakage current in Section S2.

We observe the creation of a single nanopore (i.e. fluidic channel spanning the membrane) by a sudden irreversible increase in *I_leakage_*, which is attributed to the onset of ionic current (Figure 2d and 2f) due to a discrete dielectric breakdown event. As the current continues to increase, the nanopore further enlarges (Figure 2g). We use a feedback control mechanism to rapidly terminate the trans-membrane potential when the current exceeds a pre-determined threshold, *I_cutoff_*. A threshold, set as *I_cutoff_*/*I_leakage_* <1.2, which is generally sufficient to terminate Δ*V* within ∼0.1 s of the breakdown event, can produce nanopores on the order of 2-nm in diameter as shown by the I-V curves in Figure 2 h (see Section S3 for additional results). In addition, following the nanopore fabrication event, we can continue to enlarge its size with sub-nm precision by applying moderate AC electric field square pulses in the range of ±0.2-0.3 V/nm, similar to Beamish *et al.* [23], [24]. This allows the nanopore size to be precisely tuned, for a particular sensing application, directly in neutral KCl solution.

### I-V Characteristics and Noise

To infer the nanopore size upon fabrication, we measure its ionic conductance, *G*, and relate it to an effective diameter, *d*, assuming a cylindrical geometry and accounting for access resistance [25], [26], using
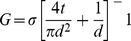
, where *σ* is the bulk conductivity of the solution. This method, practical for nanopores fabricated in liquids, provides a reasonable first order estimate of the pore size [26], [27] as confirmed by DNA translocations, and compares well with actual dimensions obtained from TEM images (see Sections S4 and S8). I-V curves are performed in a ±1 V window, where the leakage current can safely be ignored. Figure 2 h reveals an ohmic electric response in 1 M KCl. The majority of our nanopores exhibit linear I-V curves upon fabrication. The remaining nanopores that show signs of self-gating or rectification can be conditioned, by applying moderate electric field pulses [23], to slightly enlarge them until an ohmic behaviour is attained in high salt. Such I-V characteristics imply a relatively symmetric internal electric potential pore profile [28] which supports the symmetrical geometry with a uniform surface charge distribution assumed by our pore conductance model. Otherwise, one would expect significant rectification from multiple ≤1-nm fluidic paths or from a single narrow nano-crack of similar conductance, due to strong electrostatic double layer overlap. To further characterize the nanopores, we examined the noise in the ionic current by performing power spectral density measurements. Our fabrication method consistently produces nanopores with low-1/*f* noise levels, comparable to fully wetted TEM-drilled nanopores (see Section S5)[29], [30]. This may be attributed to the fact that nanopores are created directly in liquid rather than in vacuum. Thus far, we have successfully fabricated hundreds of individual nanopores ranging from 1 to 25-nm in size with comparable electrical characteristics that are stable for days, 66 of which are included in Figure 2. The success rate for fabricating a nanopore under the experimental parameters presented here is estimated at >99%.

### Dielectric Breakdown Mechanism

**Figure 3:**
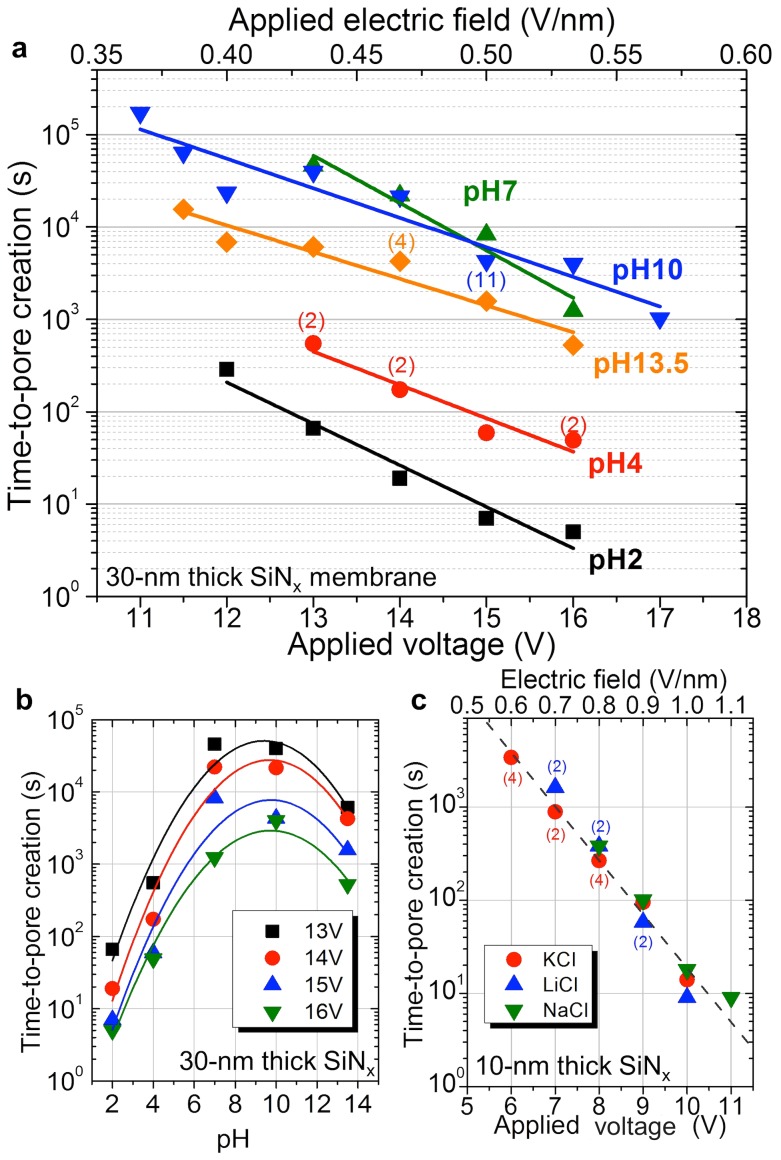
Time-to-pore creation as a function of experimental conditions. a) Semi-log plot of fabrication time of individual nanopores created in 30-nm-thick SiN_x_ membranes in 1 M KCl buffered as indicated, versus the applied voltage and the calculated applied electric field. The number of separate nanopores each data point is averaged over is indicated in parentheses. The vast majority of nanopores plotted are sub-5-nm in size (i.e. <7 nS). b) Semi-log plot of fabrication time versus pH for the data plotted in a). c) Semi-log plot of fabrication time of individual nanopores created in 10-nm-thick SiN_x_ membranes in 1 M Cl-based electrolyte buffered at pH 10 for different cationic species versus the applied voltage and the calculated applied electric field. All 66 nanopores plotted are sub-5-nm in size (i.e. <20 nS).

In order that a single, well-defined nanopore be created each time, we postulate that the leakage current must be highly localized on the insulating membrane, since for conductive substrates (semiconductors or metals) anodic oxidation leads to an array of nanopores[31]–[33]. The leakage spot(s) must also modify the membrane at the nanoscale since an aqueous KCl solution at neutral pH is not an active etchant of SiN_x_. To elucidate the mechanism leading to the formation of a nanopore, we investigate the fabrication process as a function of applied voltage, membrane thickness, electrolyte composition, concentration, and pH. Figure 3a shows the time-to-pore creation, τ, as a function of the trans-membrane potential for 30-nm-thick membranes, in 1 M KCl buffered at various pHs. Interestingly, τ scales exponentially with the applied voltage irrespective of other conditions, and can be as short as a few seconds. For a given voltage, pH has a strong effect. As seen in Figure 3b, τ can be reduced by 1,000-fold when lowering the pH from 7 to 2. We have also observed that lower salt concentrations increase the fabrication time (see Section S6). Overall, for a given fabrication condition τ is relatively consistent, though variations by a factor of 4 are common, and is uncorrelated with the size of the fabricated pore. Figure 3c shows τ for 10-nm-thick SiN_x_ membranes, buffered at pH10 in various 1 M Cl-based aqueous solutions. The fabrication time in these thinner membranes also decreases exponentially with potential, but the value required for forming a nanopore is now reduced by ∼1/3 compared to 30-nm-thick membranes, irrespective of the different cations (K^+^, Na^+^, Li^+^) tested. This observation indicates that the applied electric field in the membrane is the main driving force for initiating the fabrication of a single nanopore. Fields in the range of 0.4-1 V/nm are close to the dielectric breakdown strength of low-stress SiN_x_ films[19], and are key for intensifying the leakage current, which is thought to ultimately cause breakdown in thin insulating layers[34]. The exponential dependence of τ on potential implies the same electric field dependency, which is reminiscent of the time-to-dielectric breakdown in gate dielectrics[34]. According to the current understanding, dielectric breakdown mechanisms proceed as follows[34]–[36]: (i) probabilistic accumulation of charge traps (i.e. structural defects) by electric field-induced bond breakage or generated by charge injection from the anode or cathode, (ii) increasing up to a critical density forming a highly localized conductive path, and (iii) causing physical damage due to substantial power dissipation and the resultant heating. We propose that the process by which we fabricate a nanopore in solution is similar, though we control the damage to the nanoscale by limiting the localized leakage current, at the onset of the first, discrete breakdown event. As indicated by Figure 3, the likelihood of defect formation within the silicon nitride membrane increases with the applied voltage and the strength of the electric field. At low values, the accumulation of charge traps is accomplished with relatively low efficiency compared to the amount of charge carriers traversing the membrane, since a leakage current of tens of nanoamperes can be sustained for hours or days. Given the stochastic nature of the pore creation process, multiple simultaneous nanoscale breakdown events are unlikely. Termination of the applied voltage following the occurrence of the first breakdown event, observed by the sudden irreversible increase in *I_leakage_*, ensures that ultimately a single nanopore is created. Moreover, the fabrication of a single nanopore may result from the fact that the formation path of a nanopore experiences increased electric field strength during growth, which locally reinforces the rate of defect generation. The process by which material is removed from the membrane remains unclear, but broken bonds could be chemically dissolved by the electrolyte or following a conversion to oxides/hydrides [37], [38]. Another possibility is shearing due to localized plasticity of the membrane as a result of heating at the breakdown spot, but the efficiency of heat dissipation at the nanoscale, resulting from high surface-area-to-volume ratios, makes this less likely [22]. We explain the pH dependency on the fabrication time, for 30-nm thick SiN_x_ membranes, by the fact that breakdown at low pH is amplified by impact ionization producing an avalanche, due to the increased likelihood of hole injection or H^+^ incorporation from the anode (see Section S6 for more detail). To support the general character of nanofabrication by dielectric breakdown, we created nanopores in a different material (silicon dioxide) and present the data in Section S7.

### DNA Translocations

**Figure 4:**
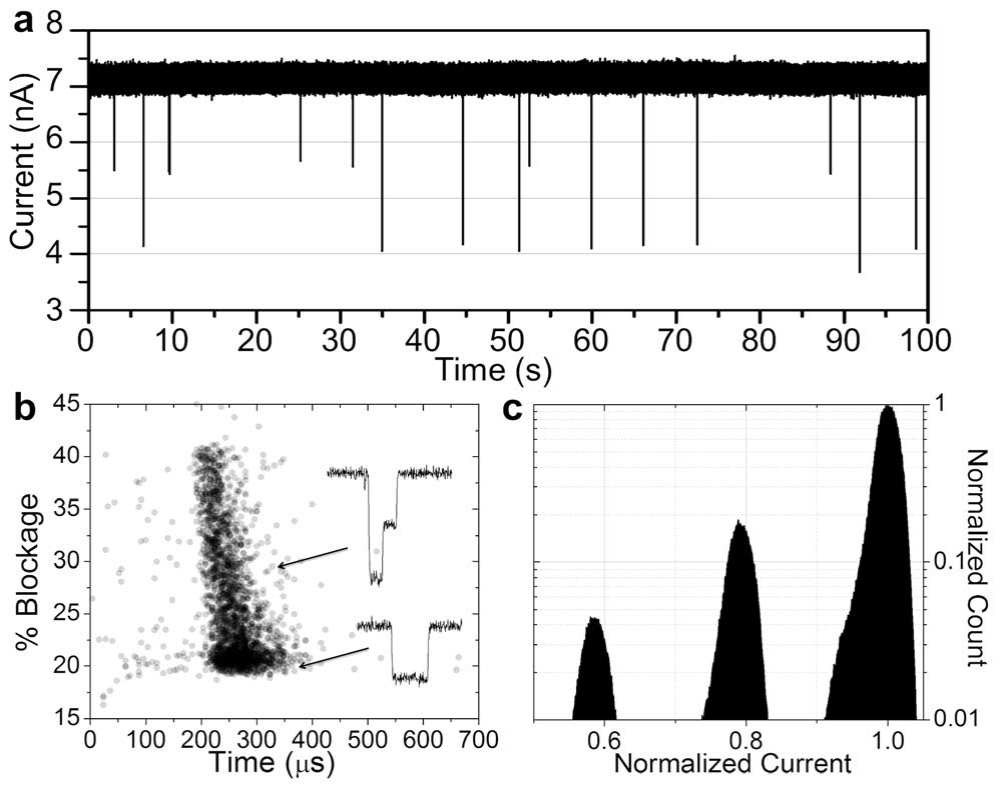
DNA Translocations. a) Ionic current trace showing multiple DNA translocation events through a ∼6.4-nm pore in a 10-nm-thick SiN_x_ membrane. Experiments performed with 10μg/mL of 5-kb DNA fragments in 3.6 M LiCl pH8, at 200 mV using an Axopatch 200B. Data sampled at 250 kHz and low-pass filtered at 100 kHz. b) Scatter plot of the normalized average current blockade (0% presenting a fully opened pore, and 100% a fully blocked pore) versus the total translocation time of a single-molecule event. Each data point represents a single DNA translocation event. The majority of the events are unfolded. There are very few anomalously long events, indicating weak DNA-pore interactions. The inset shows ionic current signatures of two single-molecule translocation events, passing in a linear and partially folded conformation. c) Histogram of the current level revealing the expected quantization of the amplitude of current blockades. Quantized levels corresponding to zero, one, two dsDNA strands in the nanopore are clearly observed.

We performed DNA translocation experiments to demonstrate that these nanopores can be leveraged for the benefit of single-molecule detection. Electrophoretically driven passage of a DNA molecule across a membrane is expected to transiently block the flow of ions in a manner that reflects the molecule length, size, charge and shape. The results using a ∼6.4-nm-diameter pore, as estimated from conductance measurements, in a 10-nm thick SiN_x_ membrane are shown in Figure 4. The scatter plot shows event duration and average current blockage of over 2,400 single-molecule translocations events of 5-kb dsDNA. The characteristic shape of the events is indistinguishable to data obtained on TEM-drilled nanopores [26], [39]–[41]. The observed quantized current blockades strongly support the presence of a single nanopore spanning the membrane. Using dsDNA (∼2.2 nm in diameter) as a molecular-sized ruler, the value of the single-level blockage events, Δ*G* = 7.4 ±0.9 nS, provides an effective pore diameter of 6.0 ±0.5-nm consistent with the size extracted from the pore conductance model [26]. This result also suggests that the membrane thickness at the vicinity of the nanopores has not been significantly altered. We observed similar DNA translocation signatures from most nanopores tested (revealing >80% success rate in detecting DNA for N = 19 nanopores tested), and provide further discussion and additional translocation data in Section S8.

### Outlook

Nanopore fabrication by controlled dielectric breakdown in solution represents a major reduction in complexity and cost over current fabrication methods, which will greatly facilitate accessibility to the field to many researchers, and provides a path to commercialize nanopore-based technologies. While we attribute the nanopore creation process to an intrinsic property of the dielectric membrane, such that the nanopore can form anywhere on the surface, our current understanding strongly suggests that the position of the pore can be determined by locally controlling the electric field strength or the material dielectric strength. This could be achieved, for instance, by nanopatterning or locally thinning the membrane, by positioning of a nanoelectrode, or by confining the field to specific areas on the membrane via micro- or nanofluidic channel encapsulation (see Section S9). The latter would also allow for the simple integration of independently addressable nanopores in an array format on a single chip.

## Materials and Methods

### Dielectric Membranes

Silicon Nitride (SiN_x_) membranes used in our experiments are commercially available as transmission electron microscope (TEM) windows (Norcada product # NT005X and NT005Z). Each membrane is made of 10-nm or 30-nm thick low-stress (<250 MPa) SiN_x_, deposited on 200-μm thick lightly doped silicon (Si) substrate by low-pressure chemical vapour deposition (LPCVD). A 50-μm × 50-μm window on the backside of the Si substrate is opened by a KOH anisotropic chemical etch. Prior to mounting into liquids, SiN_x_ membranes can be cleaned in oxygen plasma for 30 s at 30 W to facilitate wetting of the membrane surface, though this is not a requirement. All solutions used were filtered and degassed prior to use. The absence of pre-existing structural damages (e.g. pinholes, nano-cracks) is inferred by the fact that no current (<pA) is measured across a membrane at low voltages (<±1 V) prior to nanopore fabrication. The membrane side opposing the Si etch pit is the reference point for all applied voltages in this article. Silicon dioxide membranes were also purchased from TEMWindows (product# SO100-A20Q33). Note that we have also successfully fabricated nanopores on SiN_x_ membranes purchased from TEMWindows, and on custom fabricated SiN_x_ membranes.

### Instrumentation and Data Acquisition

A schematic of the experimental setup is shown in Figure 1. A silicon chip with an intact silicon nitride membrane is sandwiched between two silicone gaskets (shown in purple on the figure). It is then positioned between the two electrolyte reservoirs in a PTFE (polytetrafluoroethylene) or a PEEK (polyether ether ketone) fluidic cell. The two reservoirs filled with liquid electrolyte are electrically connected to a current amplifier by two Ag/AgCl electrodes. The entire system is encapsulated in a grounded faraday cage to isolate from electromagnetic interference. Data acquisition and measurement automation were performed using custom-designed LabVIEW software controlling a National Instruments USB-6351 or PXIe-6366 DAQ card. The value of the trans-membrane potential is set by the DAQ card. Leakage current is digitized at 250 kHz and the signal is filtered at 10 Hz. When a current exceed a pre-set threshold, the voltage bias is immediately ceased by the software (response time is ∼100 ms). I-V measurements and ionic current signal during DNA translocations are recorded using an Axopatch 200B with a 4-pole Bessel filter set at 100 kHz, with at 250 kHz sampling rate. Data analysis was carried out using custom-designed LabVIEW software to measure the duration and depth of each current blockade events.

### DNA Studies

We performed DNA translocation studies, using dsDNA fragments of 100 bp, 5 kbp, 10 kbp purchased from Fermantas (NoLimits products) in 1 M KCl pH8 or in 3.6 M LiCl pH8 at a final concentration of 10μg/mL. Lambda DNA (48.5 kbp) purchased from NewEngland BioLabs was also used.

## Supporting Information

Section S1 **Section S1 contains additional information regarding the experimental setup.**
(PDF)

Section S2 **Section S2 provides further discussion on the leakage current.**
(PDF)

Section S3 **Section S3 shows I-V curves of 8 nanopores <2.5-nm in diameter.**
(PDF)

Section S4 **Section S4 shows TEM images of nanopores fabricated by controlled dielectric breakdown.**
(PDF)

Section S5 **Section S5 presents the ionic current noise characteristics of 4 nanopores.**
(PDF)

Section S6 **Section S6 presents additional data of the time-to-pore creation versus trans-membrane potential and electric field strength.**
(PDF)

Section S7 **Section S7 demonstrates fabrication of a nanopore on a silicon dioxide membrane.**
(PDF)

Section S8 **Section S8 presents additional DNA translocation data and their analysis.**
(PDF)

Section S9 **Section S9 discusses strategies for localizing nanopores on a membrane.**
(PDF)
