# Supplementary material for: Nanopore Fabrication by Controlled Dielectric Breakdown
Source: PLoS One. 2014 Mar 21;9(3):e92880. doi: 10.1371/journal.pone.0092880 (PMC3962464; doi:10.1371/journal.pone.0092880)
Supplement: Section S1 — contains additional information regarding the experimental setup. (PDF) [file pone.0092880.s001.pdf]

Supplementary Information Section 1

S1. Procedures, and Experimental Setup

For fabricating a nanopore, a custom current amplifier is employed to measure the leakage current while applying a voltage bias of up to ±20V. Current is measured at the ground Ag/AgCl electrode with pA sensitivity. The circuit relies on simple operation-amplifiers (as shown in figure S1) to read and control voltage and current. The current signal is digitized by a data acquisition circuit, and is continuously being fed to a computer. Current is monitored in real time at a frequency of 10Hz. When the current exceeds a pre-set threshold, voltage bias is ceased immediately.

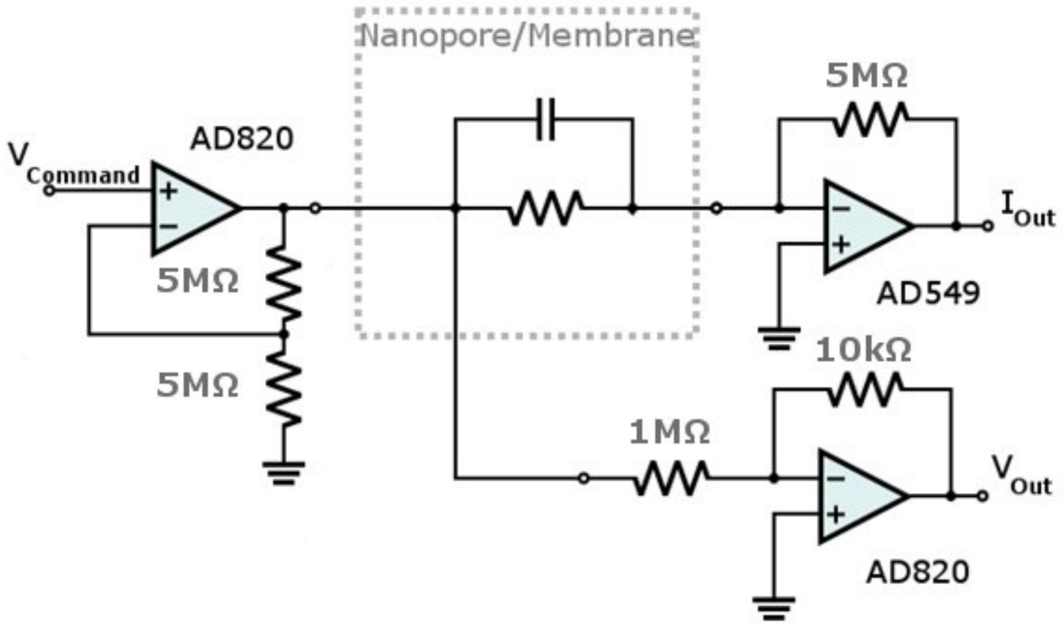

Figure S1: Schematic of the custom-built current amplifier. Op-amps used are AD820 and AD549. All op-amps are powered by a ±20V voltage source. The circuit takes in a command voltage between ±10V from a computer controlled DAQ card, which is amplified to ±20V, and sets the trans-membrane potential. Current is measured with a transimpedance amplifier topology (AD549 with a 5MΩ feedback resistor), and the signal digitized by a DAQ card. The applied trans-membrane potential is also measured. The signal is scaled by 1/100 before being digitized by a DAQ card.

After the creation of a nanopore, the custom current amplifier is replaced by a commercial amplifier, Axopatch 200B (Molecular Devices) – Figure S2. Its special

architecture allows for lower noise at higher bandwidth recording of ionic current, but can only apply trans-membrane potentials up to ±1V. Noise characterisation, *I-V* response and DNA translocation events are recorded at 250kHz sampling rate by this instrument operating at the voltage clamp mode with a 100kHz 4-pole Bessel low-pass filter.

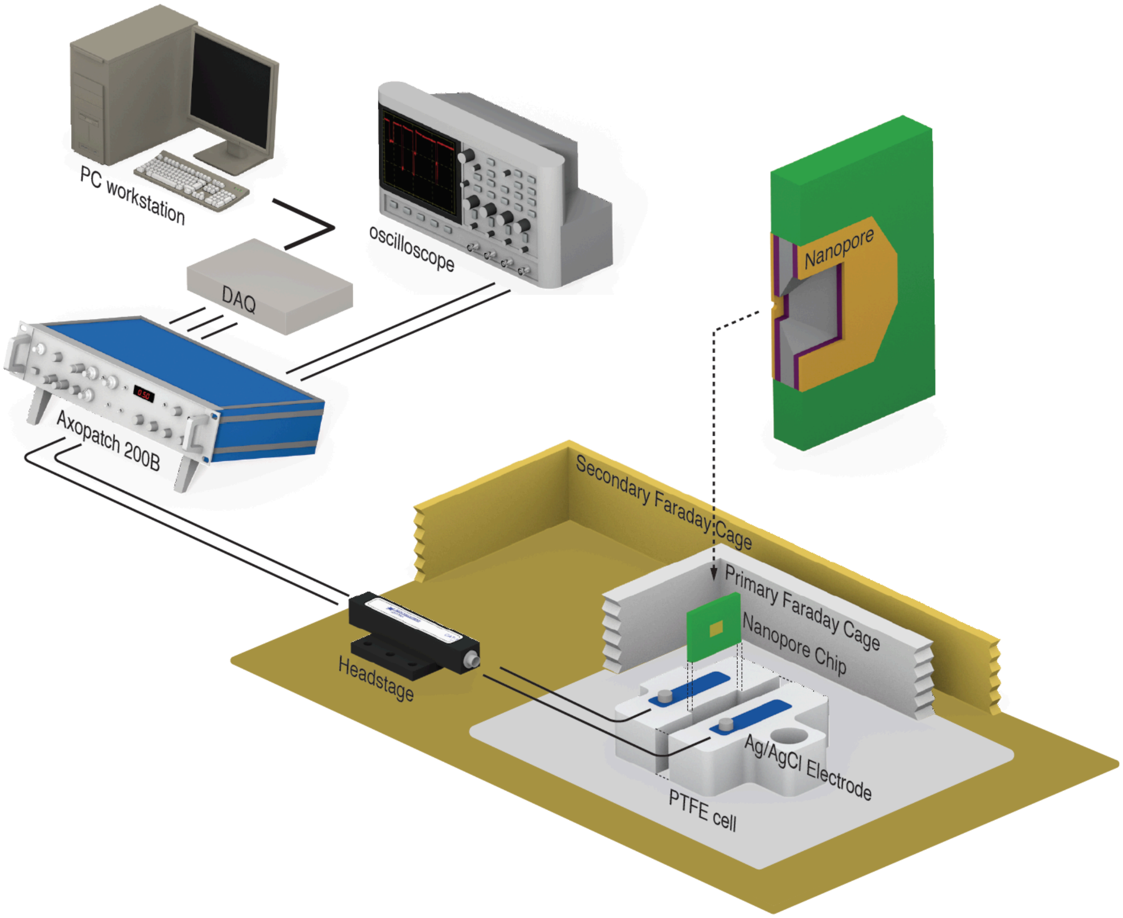

Figure S2: Schematic of the experimental setup used for DNA translocation and noise characterization. The custom current amplifier is replaced with an Axopatch 200B for low-noise high-bandwidth recordings.
