# Supplementary material for: Nanopore Fabrication by Controlled Dielectric Breakdown
Source: PLoS One. 2014 Mar 21;9(3):e92880. doi: 10.1371/journal.pone.0092880 (PMC3962464; doi:10.1371/journal.pone.0092880)
Supplement: Section S2 — provides further discussion on the leakage current. (PDF) [file pone.0092880.s002.pdf]

Supplementary Information Section 2

S2. Leakage Current

Leakage current is measured across an intact silicon nitride membrane as a function of trans-membrane potential, while immersed in an electrolyte solution (Figure 1a). At the onset of the applied voltage, a current spike from capacitive current persists for a few hundred milliseconds (proportional to the chip capacitance), followed by a slowly varying leakage current (see Figure 1f), which usually gradually increases. We note that this is not always the case, and under long-term fabrication conditions (>hours), leakage current has also been observed to be stable or decrease for different sets of experimental conditions as shown in the Figure S3.

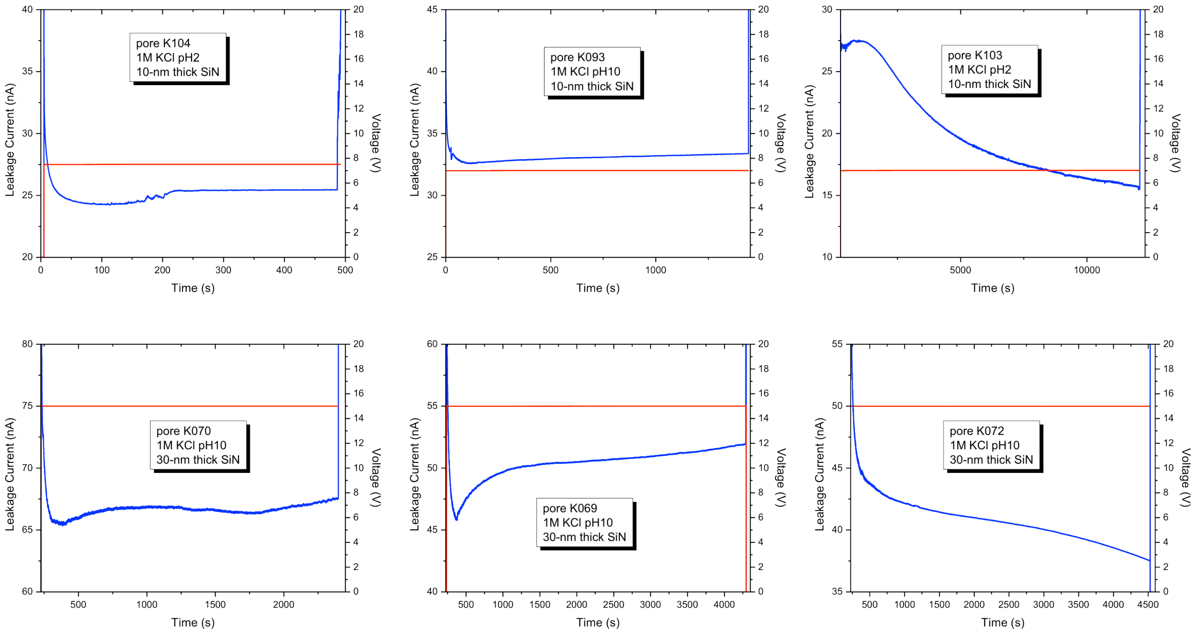

Figure S3: representative leakage current traces during nanopore fabrication for both 10-nm and 30-nm thick SiN membranes. While the leakage current often slowly changes with time, the onset of pore creation characterized by an abrupt change in current is always clearly identifiable.

This behavior is expected to be highly material dependent. The observed leakage current is attributed to trap-assisted tunneling of electrons through the silicon nitride membrane. Direct migration of ions, such as protons, is unlikely, or negligible, since the magnitude of the leakage current is not affected in a significant way by the pH of the solution, and that 30-nm thick membranes typically exhibit more leakage current for the same electric field strength, than 10-nm thick ones, as seen in Figure S3. Also note that in general the variation observed from membrane to membrane under otherwise identical conditions can

be larger than the variation observed when changing pHs from 2 to 13.5. We also argue that the slow increase in the observed leakage current corresponds to the stress induced leakage current (SILC)[1–4], which is caused by the accumulation of charge traps within the dielectric material. The measured leakage current shown in Figure 1e was recorded after 4s of an applied voltage, to remove the effect of the capacitive current spike, and avoid observing SILC. The latter generally prevents data points above 0.4V/nm to be plotted on Figure 1e since the leakage current does not reach a stable state.

Reference:

1. Kimura M, Ohmi T (1996) Conduction mechanism and origin of stress-induced leakage current in thin silicon dioxide films. Journal of Applied Physics 80: 6360. doi:10.1063/1.363655.

2. Lombardo S, Stathis JH, Linder BP, Pey KL, Palumbo F, et al. (2005) Dielectric breakdown mechanisms in gate oxides. Journal of Applied Physics 98: 121301. doi:10.1063/1.2147714.

3. Polishchuk I (2001) Physical origin of SILC and noisy breakdown in very thin silicon nitride gate dielectric. Device Research Conference. Conference Digest (Cat. No.01TH8561). IEEE. pp. 20–21. doi:10.1109/DRC.2001.937855.

4. DiMaria DJ, Cartier E (1995) Mechanism for stress-induced leakage currents in thin silicon dioxide films. Journal of Applied Physics 78: 3883.
