# Supplementary material for: Nanopore Fabrication by Controlled Dielectric Breakdown
Source: PLoS One. 2014 Mar 21;9(3):e92880. doi: 10.1371/journal.pone.0092880 (PMC3962464; doi:10.1371/journal.pone.0092880)
Supplement: Section S3 — shows I-V curves of 8 nanopores <2.5-nm in diameter. (PDF) [file pone.0092880.s003.pdf]

Supplementary Information Section 3

S3. IV curves of ~2-nm nanopores

We present, in Figure S4, I-V curves for 8 nanopores with effective diameters ranging between 1.4-nm and 2.4-nm, as calculated from our conductance-based model accounting for access resistance, to demonstrate that nanopores on the order of ~2-nm can be fabricated. The frequency response of our control software was set to 10Hz (equivalent to 100ms response time to stop the current from increasing after crossing the threshold) and the cutoff current was relatively tight (20% increase from the leakage current baseline) – Note that these settings are not particularly severe.

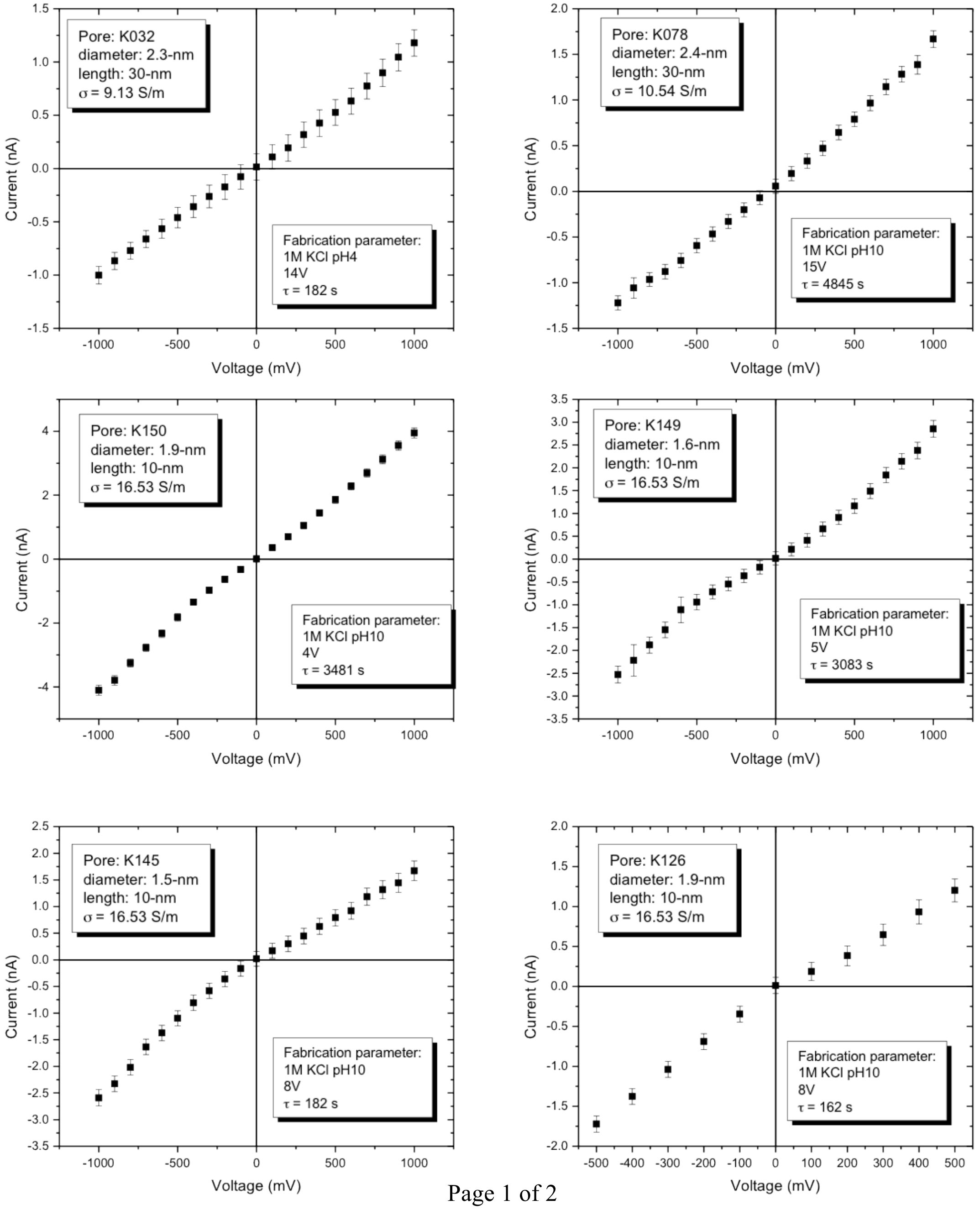

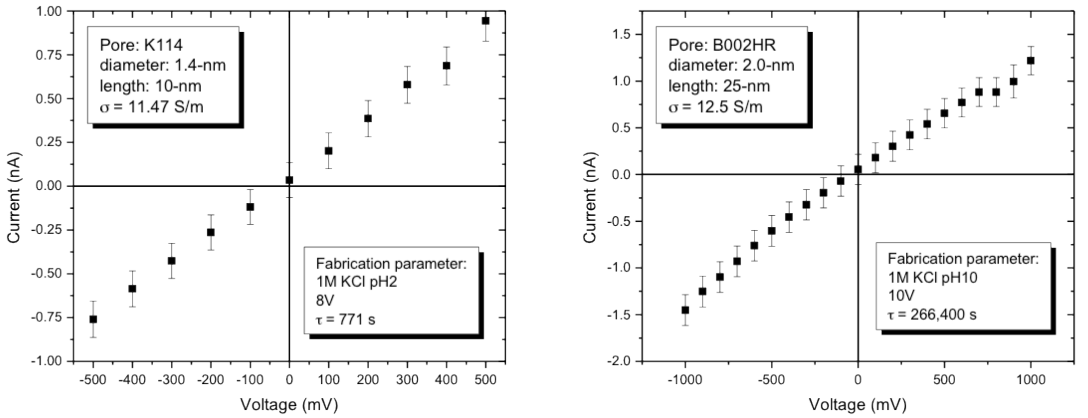

Figure S4: I-V curves of 8 independent nanopores fabricated on 10-nm and 30-nm thick SiN membranes, using different fabrication conditions.

We note that while approximations of our conductance-based model can affect the accuracy of the values extracted for the effective nanopore diameter (e.g. deviation from exact cylindrical geometry from pore to pore), data included in the manuscript and supplementary information sections generally suggest a <1-nm error.
