# Supplementary material for: Nanopore Fabrication by Controlled Dielectric Breakdown
Source: PLoS One. 2014 Mar 21;9(3):e92880. doi: 10.1371/journal.pone.0092880 (PMC3962464; doi:10.1371/journal.pone.0092880)
Supplement: Section S4 — shows TEM images of nanopores fabricated by controlled dielectric breakdown. (PDF) [file pone.0092880.s004.pdf]

Supplementary Information Section 4

S4. TEM Imaging

Imaging a nanopore fabricated by dielectric breakdown in solution under a transmission electron microscope can be a laborious task. Indeed, the nanopore creation process is an intrinsic property of the dielectric membrane, such that the nanopore can form anywhere on the surface. The commercial membranes employed in this work are 50-μm×50-μm. Looking for a sub-10-nm feature on a 25×10^2-μm^2 area is time-consuming and somewhat difficult since other debris (e.g. salt residues) can be present. Our attempts to locate and image a pore on these membranes failed.

In order to obtain TEM images of pores we therefore used a different type of membrane, with a reduced window size, graciously provided by Stratos Genomics. These custom devices have a 38-nm thick silicon nitride membrane <10-μm in size. Figure S5 shows TEM images of two independent nanopores fabricated by dielectric breakdown in solution on these custom membranes. We have observed the following three important characteristics from the nanopore images obtained thus far:

1. A top view of pores confirms a circular pore opening, which was assumed by our conductance-based model.
2. Comprehensive exploration of the membrane surface revealed the presence of a single nanopore. No other partially formed pores or unusual membrane features have been observed thus far.
3. The dimensions obtained from the TEM images are in good agreement with the size extracted from the conductance-based model, especially for pores >5-nm. Due to increased importance of surface effects (i.e. small variations of pore wall profile and surface charge density), we expect the accuracy of the conductance-based model to be lower for sub-5-nm pores in 1M KCl, particularly for thicker membranes.

These initial observations tend to indicate that the calculated pore diameter extracted from our conductance-based model may somewhat underestimate the actual diameter, though it is possible that the cleaning procedure (a few hours immersed in warm DI water) to remove salt residues may alter the pore dimensions. Nevertheless, these results support the existence of a single fluidic channel spanning the membrane, as otherwise the observed conductance would be significantly larger than what is expected from the measured TEM size of a single nanopore.

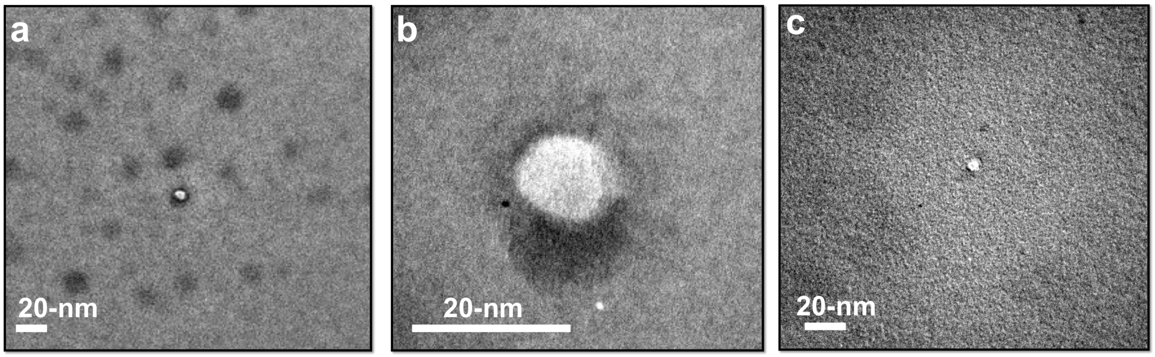

Figure S5: TEM images of nanopores fabricated by dielectric breakdown in solution. a) image of a nanopore with an estimated pore diameter extracted from conductance-based model of ~11-nm. b) Image of the same pore taken at higher magnification. Pore size measured by TEM is ~14-nm. c) Image of different nanopore on a distinct membrane with an estimated pore diameter extracted from conductance-based model of ~3-nm. Pore size measured by TEM is ~5-nm. Dark spots in the image in a) are observed on intact membranes before being immersed in solution and subjected to high electric fields. These spots are believed to be silicon rich regions, which are due to the particular LPCVD process used. Such dark spots are not observed on the commercial membranes from Norcada. The small bright spot below the pore image in b) is a defective area on the CCD, and is found on every image. Note that it is cropped from the image a).
