# Supplementary material for: Nanopore Fabrication by Controlled Dielectric Breakdown
Source: PLoS One. 2014 Mar 21;9(3):e92880. doi: 10.1371/journal.pone.0092880 (PMC3962464; doi:10.1371/journal.pone.0092880)
Supplement: Section S5 — presents the ionic current noise characteristics of 4 nanopores. (PDF) [file pone.0092880.s005.pdf]

Supplementary Information Section 5

S5. Noise Characterization

Noise is characterized by recording a time series of ionic current at a constant voltage below ±1V. We examined noise in the ionic current flowing through our fabricated nanopores, by performing power spectral density (PSD) measurements to determine their noise level. These measurements were performed using Axopatch 200B (Molecular Devices) as the current amplifier with the 4-pole Bessel filter set at 100kHz. Figure S6 shows the PSD of different membranes containing fabricated nanopores under an applied trans-membrane potential of 200mV, in 1M KCl pH8. Remarkably, the PSD for these nanopores fabricated in solution reveals low $1/f$ noise level, comparable to the best TEM-drilled nanopores[1,2], and in some instances ultra-low $1/f$ noise level can be attained, which are comparable to biological pores[1]. We noticed that, similarly to TEM-drilled pores, nanopores created by dielectric breakdown do exhibit variability in their low-frequency noise level, though low $1/f$ noise level can be obtained with very high yield (>80%). Nevertheless, in the event of a high noise nanopore, the low-frequency noise can be reduced following a conditioning procedure similar to Beamish et al.[3]. The fact that we can reliably produce nanopores with low $1/f$ noise may be attributed to the fact that our nanopores are created directly in aqueous solution and are never exposed to air, thus eliminating wetting issues, and minimizing the likelihood of nanobubbles trapped at the vicinity of a pore[4]. In addition, we speculate that the nanopore's inner wall surface can be chemically modified by the fabrication process, which may contribute to an improved noise performance.

References:

1. Tabard-Cossa V, Trivedi D, Wiggin M, Jetha NN, Marziali A (2007) Noise analysis and reduction in solid-state nanopores. Nanotechnology 18: 305505. doi:10.1088/0957-4484/18/30/305505.

2. Smeets RMM, Keyser UF, Dekker NH, Dekker C (2008) Noise in solid-state nanopores. Proceedings of the National Academy of Sciences of the United States of America 105: 417–421. doi:10.1073/pnas.0705349105.

3. Beamish E, Kwok H, Tabard-Cossa V, Godin M (2012) Precise control of the size and noise of solid-state nanopores using high electric fields. Nanotechnology 23: 405301. doi:10.1088/0957-4484/23/40/405301.

4. Smeets RMM, Keyser UF, Wu MY, Dekker NH, Dekker C (2006) Nanobubbles in Solid-State Nanopores. Physical Review Letters 97: 1–4. doi:10.1103/PhysRevLett.97.088101.

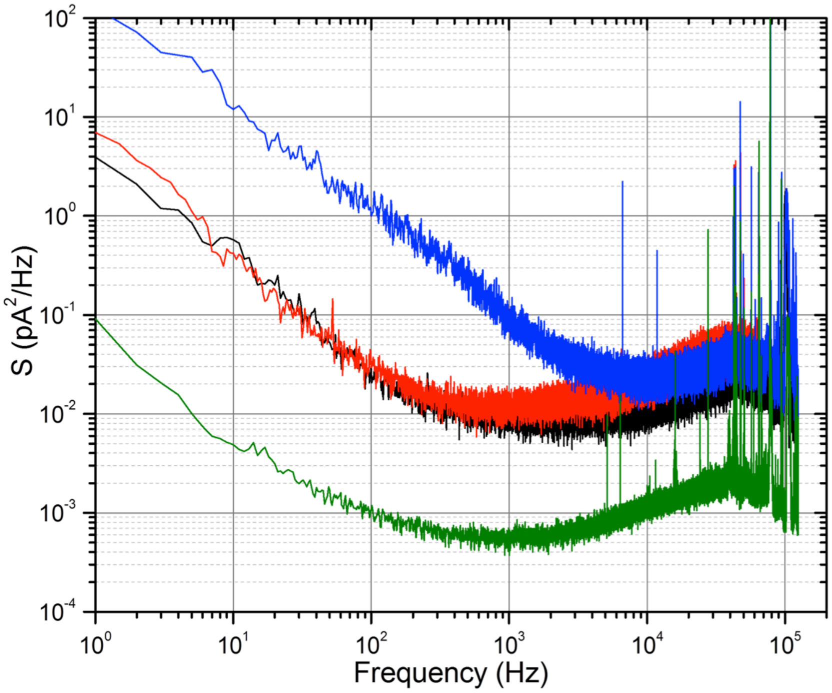

Figure S6: Power spectrum densities of the ionic current of four nanopore fabricated by controlled dielectric breakdown, recorded by an Axopatch 200B at 200mV. Data sampled at 250kHz, and low-pass filtered at 100kHz by a 4-pole Bessel filter. *Blue curve* - pore is 6-nm in diameter, created at pH2 in 1M KCl. *Red curve* - pore is 6-nm in diameter, created at pH2 in 1M KCl. *Black curve* - pore is 6-nm in diameter, created at pH2 in 1M KCl. *Green curve* - pore is 12-nm in diameter, created at pH13.5 in 1M KCl. The chip is covered with polydimethylsiloxane (PDMS) to minimize noises from membrane capacitance[1]**. At this time, there does not yet appear to be any obvious relationship between noise levels and fabrication conditions.**
