# Supplementary material for: Nanopore Fabrication by Controlled Dielectric Breakdown
Source: PLoS One. 2014 Mar 21;9(3):e92880. doi: 10.1371/journal.pone.0092880 (PMC3962464; doi:10.1371/journal.pone.0092880)
Supplement: Section S6 — presents additional data of the time-to-pore creation versus trans-membrane potential and electric field strength. (PDF) [file pone.0092880.s006.pdf]

Supplementary Information Section 6

S6. Time-to-pore creation versus Trans-membrane potential and Electric Field strength.

The fabrication of a nanopore in a dielectric membrane is similar in nature to the time-dependent dielectric breakdown (TDDB) process in solid-state devices. It is therefore interesting to show the time required to create a single nanopore in relation to the applied electric field and voltage. Here we re-plot some of the data showed in Figure 2. We tested two membrane thickness values (t = 10-nm, 30-nm), at various pH and applied voltages, V. The applied electric field is given by $E = V/t$. Figure S7 shows an approximate linear relationship in semi-log scale for the time-to-pore fabrication versus the applied voltage or applied field. This trend is reminiscent to dielectric breakdown models for solid-state devices[1,2]. However, fabrication times for the two different membrane thicknesses studied do not always follow the same behaviour. In alkaline conditions, 10-nm and 30-nm thick membrane collapse on a continuous curve as a function of applied electric field, indicating that the field strength dominates the fabrication process. In acidic conditions however, 10-nm membranes require a much higher field to create a nanopore for a given fabrication time. We speculate that the strong pH dependence observed in 30-nm thick membranes is a consequence of the greater thickness of the membrane, enabling impact ionization effects to take place. In 30-nm thick membrane, we therefore argue that breakdown at low pH is amplified by impact ionization-induced avalanche, due to the increased likelihood of H^+ incorporation or hole injection from the anode side of the membrane[3]. Nevertheless, the possibility of pH and voltage driven chemical and electrochemical reactions participating in the pore fabrication process should not be ignored and could also be playing a role in the observed results.

Figure S7 also contains data points at the same pH7, but at two different salt concentration (1M vs. 10 mM KCl), showing that fabrication time is reduced in high salt compared to low salt.

References:

1. Lombardo S, Stathis JH, Linder BP, Pey KL, Palumbo F, et al. (2005) Dielectric breakdown mechanisms in gate oxides. Journal of Applied Physics 98: 121301. doi:10.1063/1.2147714.

2. Cui H, Burke P a. (2004) Time-dependent dielectric breakdown of hydrogenated silicon carbon nitride thin films under the influence of copper ions. Applied Physics Letters 84: 2629. doi:10.1063/1.1703839.

3. Albella JM, Montero I, Martinez-Duart JM (1987) A THEORY OF AVALANCHE BREAKDOWN OXIDATION DURING. Electrochimica Acta 32: 255–258.

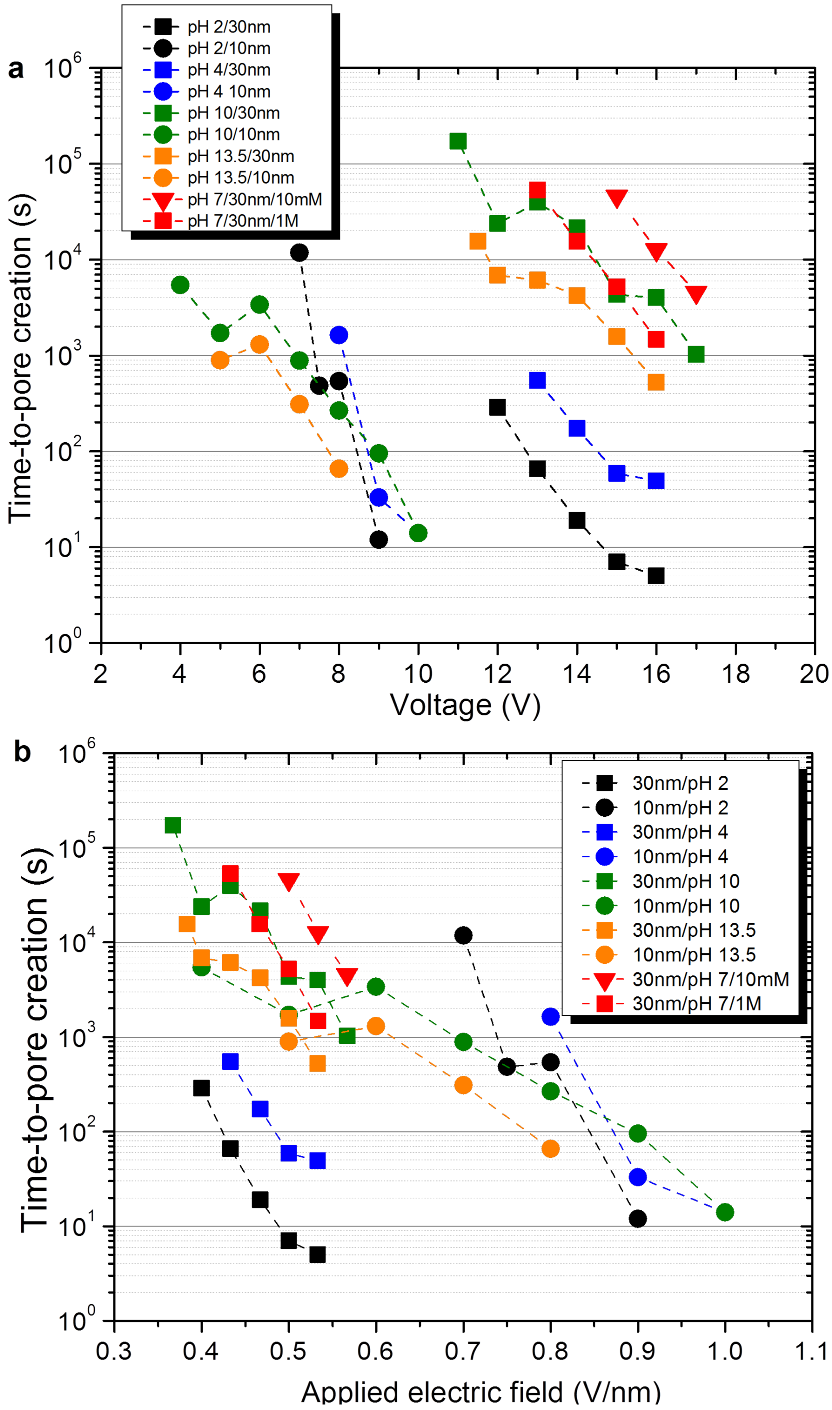

Figure S7: Time-to-pore creation a nanopore plotted in a log scale against a) applied voltage, b) calculated electric field strength, for 10-nm and 30-nm thick silicon nitride membranes, in 1M KCl at various pHs, except for three points in 10mM KCl pH7. Some of this data is plotted in Figure 2. Some data points are averaged over multiple creation events.
