# Supplementary material for: Nanopore Fabrication by Controlled Dielectric Breakdown
Source: PLoS One. 2014 Mar 21;9(3):e92880. doi: 10.1371/journal.pone.0092880 (PMC3962464; doi:10.1371/journal.pone.0092880)
Supplement: Section S7 — demonstrates fabrication of a nanopore on a silicon dioxide membrane. (PDF) [file pone.0092880.s007.pdf]

Supplementary Information Section 7

S7. Fabrication on SiO_2 Membranes.

We fabricated nanopores in 20-nm thick SiO_2 membranes purchased from SIMPore (TEMWindows – product# SO100-A20Q33). A weaker electric field strength was needed to fabricate a nanopore in SiO_2 in a given time compared to SiN, which we attribute to the reduced dielectric constant, and is consistent with dielectric breakdown mechanism for the pore formation. Figure S8 shows the nanopore creation event during a 2 s pulse at 7.5V and continued enlargement at 8V, as well as the resulting I-V curve of the nanopore, showing an effective diameter of 6.2-nm. We successfully confirmed the ability of the nanopore to detect DNA by studying the translocation of 10-kpb dsDNA molecules.

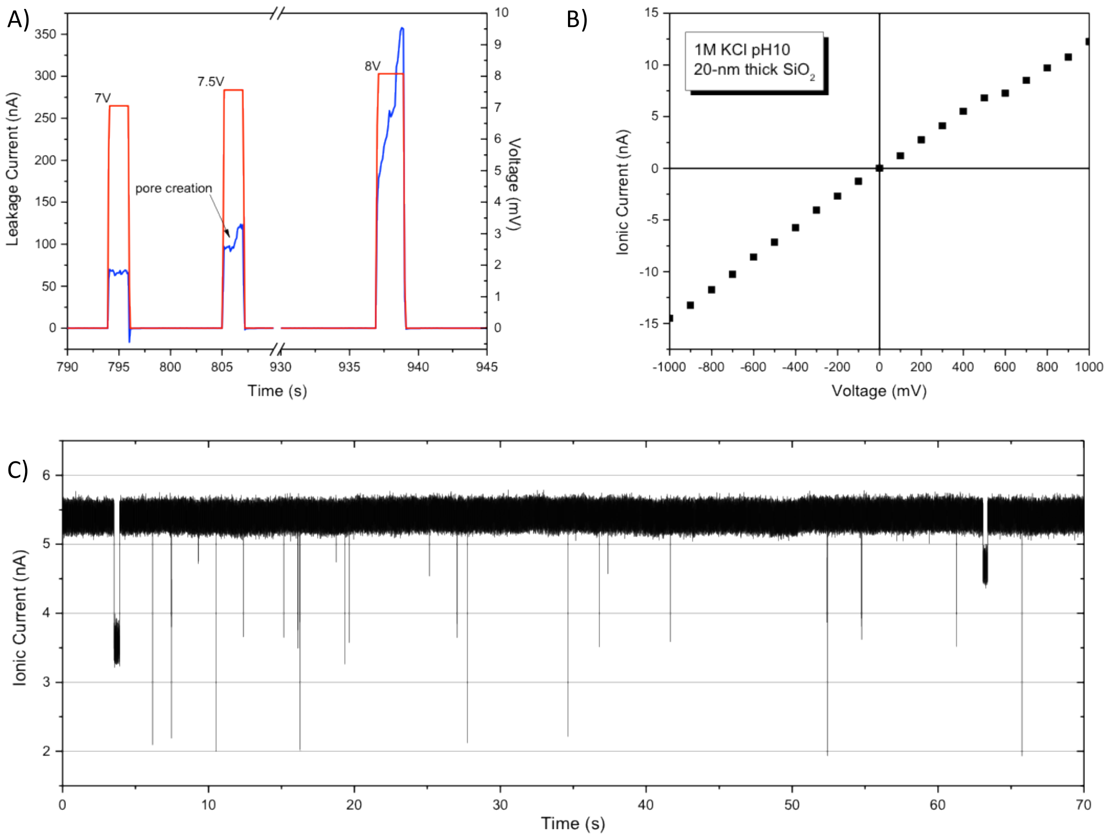

Figure S8: A) Leakage current versus time for different strength of applied voltage pulses. Pore creation happens during the 2s pulse at 7.5V, and is enlarged during the subsequent 8V pulse. B) I-V curve of the resulting nanopore, with an effective diameter of 6.2-nm. C) Ionic current trace at 400mV in 1M LiCl pH8, showing characteristics blockages from translocating 10kpb dsDNA molecules.
