# Supplementary material for: Nanopore Fabrication by Controlled Dielectric Breakdown
Source: PLoS One. 2014 Mar 21;9(3):e92880. doi: 10.1371/journal.pone.0092880 (PMC3962464; doi:10.1371/journal.pone.0092880)
Supplement: Section S8 — presents additional DNA translocation data and their analysis. (PDF) [file pone.0092880.s008.pdf]

Supplementary Information Section 8

S8. DNA Translocation

The ability to detect single molecule events, such as DNA translocation, is an essential feature of a nanopore-based sensing device. To illustrate that nanopores fabricated by our method are compatible for single bio-molecule sensing applications, we performed a systematic investigation of 18 independent nanopores fabricated in a wide range of experimental conditions in 10-nm thick silicon nitride membranes. 11 nanopores were between 4.5-nm and 6.5-nm in effective size, 4 nanopores were between 7-nm and 9-nm in effective size, and 3 nanopores were between 10-nm and 25-nm in effective size. These nanopores were systematically tested by injecting 5-kb dsDNA fragments (Fermentas, NoLimits) on one side of the membrane in 3.6M LiCl pH8 at a final concentration of 10μg/mL. LiCl was chosen to maximize the current blockades and increase translocation times[1]. Amongst the 18 pristine membranes used, all created a nanopore, while 15 of them successfully showed DNA translocation events, and 9 of them detected >1,000 events before clogging[*]. In other DNA translocation studies, we have also demonstrated detection of 100-bp DNA fragments, 10-kb DNA fragments, Lamda DNA molecules, and 10-nm and 30-nm thick SiN_x membranes, and 20-nm thick SiO_2 membranes. The yield is comparable to if not greater than TEM-drilled nanopores (based on our experience and from discussions with other groups). However, we should point out that these statistics were obtained without any optimization in the fabrication conditions, and we therefore believe that major improvement can be achieved.

Below, we present additional DNA translocation results of a 6.5-nm pore in a 10-nm thick silicon nitride membrane (fabrication parameters: 7V, 1M KCl pH2, τ=11,837s), in 3.6M LiCl buffered at pH8, at five different voltages ranging between 200mV and 1000mV. Current traces showing some of these events are shown in Figure S9.

[*] We note that clogged pores can often be unclogged by following a process similar to Beamish et al.[11], though at the expense of an increase in size, which can nevertheless be minimized to a few nanometers.

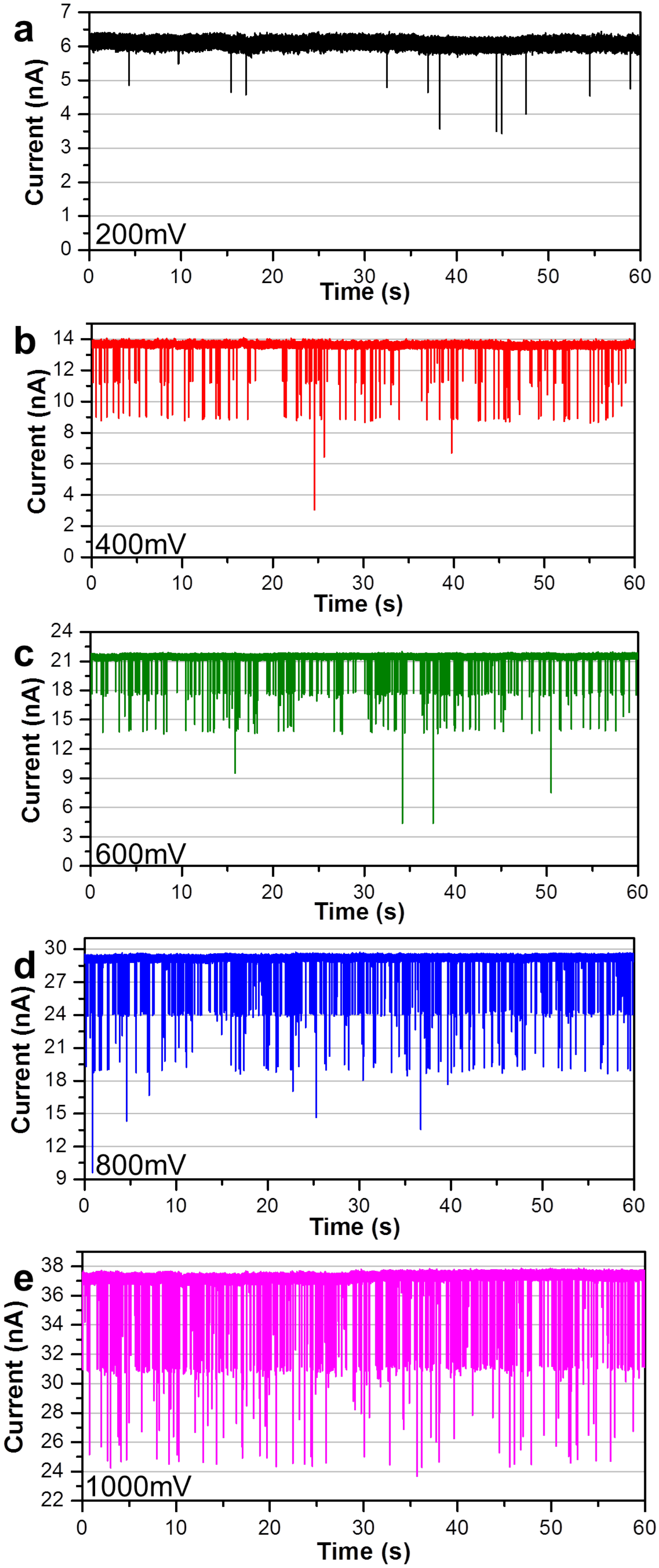

Figure S9: Current versus time of 5-kbp dsDNA translocation events for a 6.5-nm pore in a 10-nm membrane in pH 8 3.6M LiCl. Data recording at 250kHz sampling rate, and low-pass filtered by a 4-pole Bessel filter set at 100kHz. a) Data at 200mV was recording when the nanopore had an effective size of 6.0-nm. The pore partially clogged after a few hundreds events. A few pulses of moderate electric fields (0.2 V/nm) were used to unclogged it, which also increased its effective size to 6.5-nm. b-e) Data at 400mV, 600mV, 800mV and 1000mV was subsequently recorded without flushing out or adding new DNA molecules.

Expected Conductance Blockades – These current traces, with observed blockades showing multiple quantized ionic current levels, are characteristic of long negatively charged polymers electrophoretically forced through a nanopore and compare qualitatively well with published results obtained on TEM-drilled nanopores[2–4]. The interpretation that the observed current blockades are indeed due to dsDNA translocation through nanopores, fabricated by our method, is supported by both the increase in current blockage amplitude and the decrease in the mean translocation time with increasing trans-membrane potential across the nanopore (see also Figures S10 and S11). The expected current blockage from a dsDNA molecule translocating through a nanopore will depend on the particular geometry of the nanopore, and the electrolyte composition[5]. Translocation data obtained from nanopore fabricated by controlled dielectric breakdown compares very well with results obtained from TEM-drilled pores. Similar to what has been previously done for TEM-drilled pores, our results can be rationalized by assuming a cylindrical nanopore geometry, from which we can approximate the expected conductance blockade, ΔG, from a single strand of dsDNA spanning the length of the pore as[2,6]:

$$\Delta G = \frac{\sigma \pi d_{DNA}^2}{4L} \quad \text{(S1)}$$

where σ is the solution conductivity, and L the pore length. This simple expression based on a cylindrical model of the pore is frequently used in the nanopore literature[7] and allows L to be determined without a priori knowledge of the pore diameter. In the limit of high salt concentration (3.6M LiCl, used in this case) and small nanopores (<10-nm), edge effects become less important due to both increased effective shielding of the electrolyte and reduced relative importance of the access resistance as compared to pore resistance (see Figure 1 of Kowalczyk et al.[7]). We therefore argue that the approximations of equation (S1) affect only slightly the accuracy of our results (see the Table1 below, comparing the various values obtained with different models).

Solving equation (S1) assuming a diameter of B-form double-stranded DNA of 2.2 nm, and taking the effective pore length as the nominal membrane thickness (10-nm), we obtain ΔG = 6.3 nS for the experimental conditions used in Figure S12-S13 (conductivity of 16.45 S/m). Our experimental results, of ΔG = 6.2 ± 0.2nS, for the various trans-membrane potentials tested are therefore consistent with a first-order cylindrical approximation of the nanopore geometry.

Calculation of the effective pore length – DNA translocation results can be used to verify that the membrane thickness has not been thinned in a noticeable way during the

nanopore fabrication process. We can calculate the effective pore length at the pore location using equation (S1) and compare it to the nominal membrane thickness.

We have performed an in-depth analysis of 8 independent DNA translocation experiments on 10-nm thick silicon nitride membranes, totaling ~30,000 events showing unfolded conductance blockades, and using a range of fabrication conditions. Table 1 and Table 2 summarize our results, which show an average effective pore length, L = 9.0 ± 0.3nm. This value is within the tolerance range of the manufacturer of these commercial membranes, quoted as a thickness of 10 ± 1nm (product #: Norcada NT005Z).

Table 1: Translocations were performed in 3.6M LiCl pH 8, with 5kbp dsDNA, except pore K097 which used 10kpb dsDNA. Nominal membrane thickness is 10-nm.

Pore ID	$V(mV)$	$G(nS)$	$\Delta G(nS)$	L (nm)	d (nm)	d_{simple} (nm)	# of events
**K093** *(pore shown in text)*	200	35.4 ± 0.6	7.4 ± 0.9	8 ± 1	6.0 ± 0.5	4.8	2490
	400	33.8 ± 0.3	6.6 ± 0.6	9 ± 1	6.1 ± 0.5	5.0	190
	600	36.4 ± 0.3	7.1 ± 0.5	9 ± 1	6.2 ± 0.5	5.0	549
**K097**	200	23.0 ± 0.4	8.7 ± 0.6	7.2 ± 0.9	4.3 ± 0.3	3.6	277
	400	25.1 ± 0.3	8.9 ± 0.4	7.0 ± 0.8	4.5 ± 0.3	3.7	166
	600	25.3 ± 0.1	8.3 ± 0.1	7.5 ± 0.8	4.7 ± 0.3	3.8	346
	1000	27.1 ± 0.2	8.8 ± 0.3	7.1 ± 0.7	4.8 ± 0.4	3.9	384
**K099**	200	25.3 ± 0.6	5.6 ± 0.9	11 ± 2	5.5 ± 0.5	4.7	722
	400	27.7 ± 0.3	6.0 ± 0.4	10 ± 1	5.6 ± 0.4	4.7	2172
	600	28. ±0.2	6.4 ± 0.3	10 ± 1	5.6 ± 0.4	4.7	1724
**K103** *(pore shown in supp. info)*	200	29.8 ± 0.6	5.7 ± 0.9	11 ± 2	6.0 ± 0.6	5.0	161
	400	34.1 ± 0.1	6.0 ± 0.2	10 ± 1	6.4 ± 0.5	5.2	2867
	600	35.8 ± 0.4	6.4 ± 0.5	10 ± 1	6.4 ± 0.5	5.2	4117
	800	36.5 ± 0.2	6.3 ± 0.2	10 ± 1	6.5 ± 0.5	5.3	3408
	1000	37.3 ± 0.1	6.4 ± 0.1	10 ± 1	6.6 ± 0.5	5.3	747
**K104**	200	32.3 ± 0.6	7.0 ± 0.9	9 ± 2	5.8 ± 0.5	4.7	120
	400	32.4 ± 0.3	7.1 ± 0.4	9 ± 1	5.8 ± 0.4	4.7	879
	600	32.9 ± 0.3	7.1 ± 0.4	9 ± 1	5.8 ± 0.4	4.7	1247
	800	33.1 ± 0.2	7.3 ± 0.5	9 ± 1	5.8 ± 0.4	4.7	1070
	1000	33.9 ± 0.2	7.1 ± 0.4	9 ± 1	5.9 ± 0.4	4.8	1176
**K106**	200	43.7 ± 0.9	6 ± 1	11 ± 3	7.6 ± 0.8	6.1	175
	1000	46.6 ± 0.2	5.7 ± 0.2	11 ± 1	7.9 ± 0.6	6.3	83
**K123**	400	32.9 ± 0.2	8.1 ± 0.4	7.7 ± 0.8	5.5 ± 0.4	4.4	1119
	600	33.3 ± 0.3	8.4 ± 0.4	7.4 ± 0.8	5.5 ± 0.4	4.4	839

	800	34.6 ± 0.3	8.1 ± 0.3	7.7 ± 0.8	5.7 ± 0.4	4.5	2798
	1000	35.1 ± 0.2	8.2 ± 0.2	7.6 ± 0.8	5.7 ± 0.4	4.6	586
**K124**	200	32.9 ± 0.6	9 ± 1	7 ± 1	5.4 ± 0.5	4.3	496
	400	31.4 ± 0.3	8.2 ± 0.5	7.6 ± 0.9	5.4 ± 0.4	4.3	1451
	600	33.9 ± 0.2	8.6 ± 0.3	7.3 ± 0.8	5.5 ± 0.4	4.4	849
	800	34.6 ± 0.2	8.4 ± 0.3	7.4 ± 0.8	5.6 ± 0.4	4.5	1203
	1000	35.6 ± 0.1	8.1 ± 0.1	7.7 ± 0.8	5.8 ± 0.4	4.6	69

Note that:

- **K093 was fabricated in pH 10 1M KCl at -9V for 360s, and conditioned at ±3V AC in pH 8 3.6M LiCl for 800s.**
- **K097 was fabricated in pH 10 1M KCl at -8V for 258s and conditioned at ±3V AC in pH 8 3.6M LiCl for 3900s.**
- **K099 was fabricated in pH 2 1M KCl at -8V for 300s and conditioned at ±3V AC in pH 8 3.6M LiCl for 4634s.**
- **K103 was fabricated in pH 2 1M KCl at -7V for 11837s and conditioned at ±4V AC in pH 8 3.6M LiCl for 4500s.**
- **K104 was fabricated in pH 2 1M KCl at -7.5V for 482s and conditioned at ±3V AC in pH 8 3.6M LiCl for 8884s.**
- **K106 was fabricated in pH 13.5 1M KCl at -8V for 66s and conditioned at ±3V AC in pH 8 3.6M LiCl for 2360s.**
- **K123 was fabricated in pH 10 1M NaCl at -10V for 18s and conditioned at ±3V AC in pH 8 3.6M LiCl for 1270s.**
- **K124 was fabricated in pH 10 1M NaCl at -9V for 101s and conditioned at ±3V AC in pH 8 3.6M LiCl for 540s**

Error on the calculated membrane thickness was determined as follow:

$$\delta \mathrm{L} = \frac{A_{DNA}}{\Delta G}\sqrt{\delta\sigma^2 + \frac{\sigma^2 \delta \Delta G^2}{\Delta G^2}}$$

where error on the solution conductivity was estimated at 10%, and error on the conductance blockade is taken as the square root of the sums of the squares of the HWHMs histograms of the first blocked state and open pore state.

We then use this value of effective membrane thickness L to refine our pore diameter d using open pore conductance $G = \sigma\left(\frac{4L}{\pi d^2} + \frac{1}{d}\right)^{-1}$, obtaining

$$d = \frac{G}{2\sigma}(1+K) \pm \sqrt{\left(\frac{1+K}{2\sigma} - \frac{4L}{\pi G K}\right)^2 \delta G^2 + \left(\frac{4L}{\pi\sigma K} - \frac{G}{2\sigma^2}(1+K)\right)^2 \delta\sigma^2 + \left(\frac{4}{\pi K}\right)^2 \delta L^2}$$

where $K = \sqrt{1 + \frac{16\sigma L}{\pi G}}$. All errors are rounded to one significant figure, though appropriate rounding is only done after the full analysis has been completed. Average error is $\frac{1}{n}\sqrt{\sum \Delta L_i^2}$ where n is the number of voltage levels at each pore or the number of pores considered, as appropriate.

For the sake of comparison, we have included d_{IV}, the value of the diameter we measured from the conductance prior to starting the DNA experiment in question (assuming a nominal membrane thickness of 10-nm), as well as d_{simple}, which is the value of diameter calculated without access resistance from the conductance blockage according to the equation:

$$\frac{\Delta G}{G} = \frac{d_{DNA}^2}{d_{simple}^2}$$

Table 2: Averaged values of each nanopore compared to the size extracted from I-V measurements, which assumed a nominal membrane thickness of 10-nm.

Name	$L_{average}(nm)$	$d_{average}(nm)$	$d_{IV}(nm)$
**K093**	8.9 ± 0.7	6.1 ± 0.3	6.4
**K097**	7.2 ± 0.4	4.6 ± 0.2	5.3
**K099**	10.5 ± 0.9	5.6 ± 0.3	5.3
**K103**	10.2 ± 0.6	6.4 ± 0.2	5.9
**K104**	8.8 ± 0.5	5.8 ± 0.2	6.1
**K106**	11 ± 1	8 ± 1	7.5
**K123**	7.6 ± 0.4	5.6 ± 0.2	6.2
**K124**	7.5 ± 0.4	5.5 ± 0.2	6

Analysis of the DNA data indicates that the membrane thickness at the nanopore location is not particularly thinned by the pore creation process. As importantly, it strongly supports our assumed cylindrical pore geometry as the measured membrane thickness and the nominal membrane thickness generally match within error. This observation is supported by the TEM images shown in Figure S5, revealing a circular opening of the nanopore, consistent with the cylindrical pore model, and not showing signs of membrane thinning around the pore.

Quantized Conductance Levels – Further analysis of the translocation data reveals the presence of quantized levels in many blockade events arising from integer multiples of the number of dsDNA strands present in the pores[2,8]. The equidistant separation of the blockade levels of Figure S9 is in agreement with the assumption that the current blockade is proportional to the total cross-sectional area of dsDNA molecules. In addition, we have analyzed each DNA event to measure the average current blockade and their duration. Figure S10 is the resulting scatter plot of the detected translocation events.

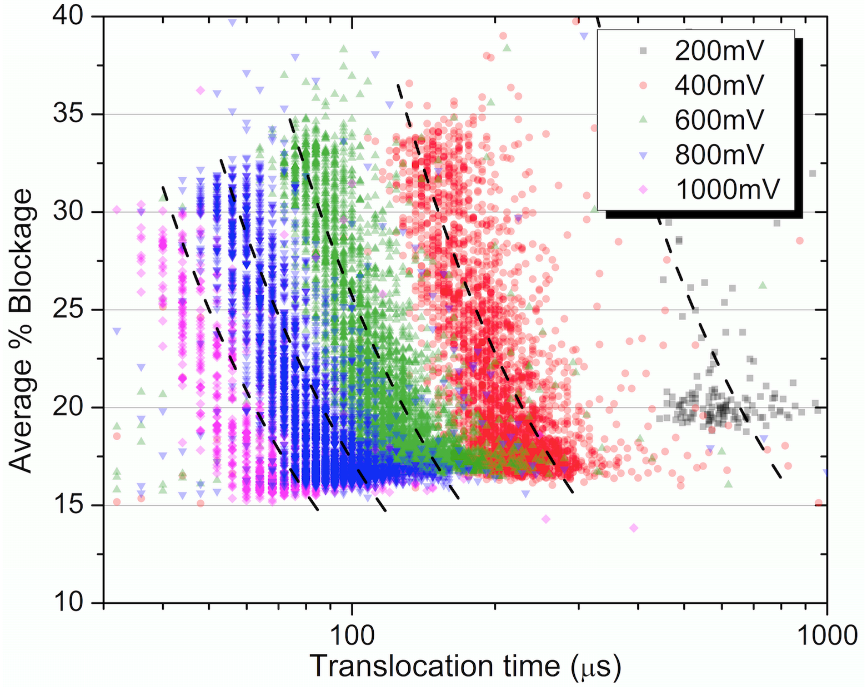

Figure S10: Scatter plot of normalized ionic current blockade amplitude versus event duration of 5kbp dsDNA translocation events for a 6.5-nm pore in a 10-nm membrane in pH 8 3.6M LiCl. The different behavior at 200mV reflects the fact the pore was ~6-nm and partially clogged after a few hundreds events. A few pulses of moderate electric fields of 0.2V/nm were used unclogged it, which also increased its size to ~6.5-nm. Also note that at 200mV DNA mostly traversed in an unfolded fashion (linear). Data recording at 250kHz sampling rate with a 100kHz 4-pole Bessel filter. The experiments lasted many hours in total. 200mV dataset contains 161 events, 400mV dataset contains 2867 events, 600mV dataset contains 4117 events, 800mV dataset contains 3408 events, and 1000mV dataset contains 747 events. Note that the conductance blockade amplitude at these voltages is slightly reduced, which is also indicative of a larger pore.

The data agrees reasonably well with the equivalent charge deficit (e.c.d) curve[8,9], which implies that friction from the wall of a pore is not significant under the conditions tested. Also note that anomalously long DNA translocation events are rare.

The mean translocation time from single-level (unfolded) events at a given trans-membrane potential is extracted by fitting to a Gaussian the distribution to the translocation times. The average DNA velocity is calculated by dividing the length of the DNA by the mean translocation time. Figure S11 shows the mean translocation time and the averaged velocity as a function of applied voltage. The mean translocation time data scale roughly as ~$1/V$ as expected[6]. The calculated DNA velocities are in accordance with published results[10], though as with TEM-drilled nanopores we have observed large variability between nanopores.

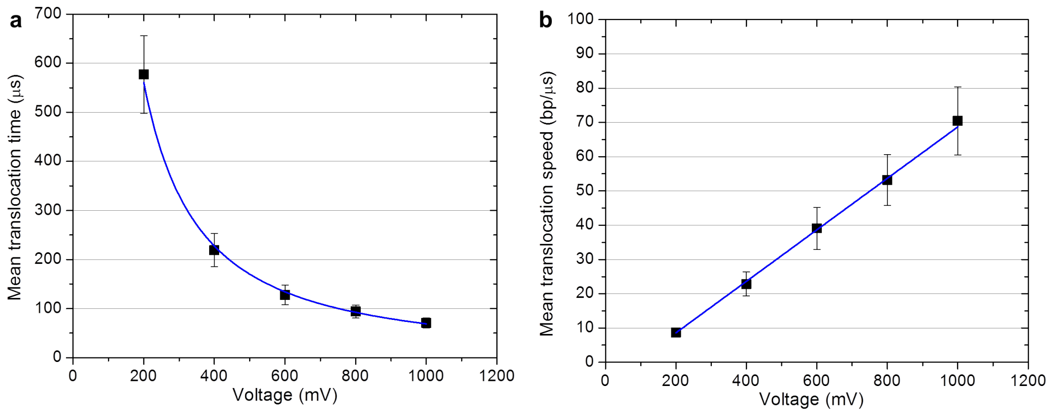

Figure S11: a) Mean translocation time (dwell time) of single level events (unfolded) versus applied voltage, showing a ~1/V dependence. b) Calculated velocity (*v* = 5-kbp/dwell time), showing the speed is linear with voltage.

Finally, the fact that some translocation events characterized 2 and even 3 quantized blockage levels, the latter representing more than 50 % blockage amplitude, strongly supports the conclusion that only a single ~6-nm nanopore is spanning the membrane. If two, or more, nanopores existed on the membrane, considering the size of dsDNA (~2.2nm), 2 and 3 blockade levels could not be achieved, since the area of a single ~6-nm nanopore can be equivalent to two ~4-nm nanopores (assuming cylindrical geometry), and the likelihood of consistently synchronizing translocation events between pores would be low.

Overall, our method fabricates single nanopores, with very high yield, which generate electrical signals from individual translocating DNA molecules that are virtually indistinguishable from TEM-drilled pores. This experimental fact strongly supports the assumption that our fabrication method produces, to first-order, cylindrical channels spanning the membrane, as opposed to long tortuous path or very narrow slits across the membrane. This is further supported by the TEM images obtained of these fabricated pores.

References:

1. Kowalczyk SW, Wells DB, Aksimentiev A, Dekker C (2012) Slowing down DNA translocation through a nanopore in lithium chloride. Nano letters 12: 1038–1044. doi:10.1021/nl204273h.

2. Storm a., Chen J, Zandbergen H, Dekker C (2005) Translocation of double-strand DNA through a silicon oxide nanopore. Physical Review E 71: 1–10. doi:10.1103/PhysRevE.71.051903.

3. Bo L, Albertorio F, Hoogerheide DP, Golovchenko JA, Lu B (2011) Origins and consequences of velocity fluctuations during DNA passage through a nanopore. Biophysical journal 101: 70–79. doi:10.1016/j.bpj.2011.05.034.

4. Wanunu M, Dadosh T, Ray V, Jin J, McReynolds L, et al. (2010) Rapid electronic detection of probe-specific microRNAs using thin nanopore sensors. Nature Nanotechnology 5: 807–814. doi:10.1038/nnano.2010.202.

5. Frament CM, Dwyer JR (2012) Conductance-Based Determination of Solid-State Nanopore Size and Shape: An Exploration of Performance Limits. The Journal of Physical Chemistry C 116: 23315–23321. doi:10.1021/jp305381j.

6. Fologea D, Uplinger J, Thomas B, McNabb DS, Li J (2005) Slowing DNA translocation in a solid-state nanopore. Nano letters 5: 1734–1737. doi:10.1021/nl051063o.

7. Kowalczyk SW, Grosberg AY, Rabin Y, Dekker C (2011) Modeling the conductance and DNA blockade of solid-state nanopores. Nanotechnology 22: 315101. doi:10.1088/0957-4484/22/31/315101.

8. Li J, Gershow M, Stein D, Brandin E, Golovchenko J a (2003) DNA molecules and configurations in a solid-state nanopore microscope. Nature materials 2: 611–615. doi:10.1038/nmat965.

9. Fologea D, Brandin E, Uplinger J, Branton D, Li J (2007) DNA conformation and base number simultaneously determined in a nanopore. Electrophoresis 28: 3186–3192.

10. Venkatesan BM, Bashir R (2011) Nanopore sensors for nucleic acid analysis. Nature nanotechnology 6: 615–624. doi:10.1038/NNANO.2011.129.

11. Beamish E, Kwok H, Tabard-Cossa V, Godin M (2012) Precise control of the size and noise of solid-state nanopores using high electric fields. Nanotechnology 23: 405301. doi:10.1088/0957-4484/23/40/405301.
