# Supplementary material for: Nanopore Fabrication by Controlled Dielectric Breakdown
Source: PLoS One. 2014 Mar 21;9(3):e92880. doi: 10.1371/journal.pone.0092880 (PMC3962464; doi:10.1371/journal.pone.0092880)
Supplement: Section S9 — discusses strategies for localizing nanopores on a membrane. (PDF) [file pone.0092880.s009.pdf]

Supplementary Information Section 9

S9. Strategies to Localize the Pore Creation on the Membrane.

As mentioned in the last paragraph of the manuscript, we suspect that the nanopore creation process to be an intrinsic property of the dielectric membrane, such that a nanopore can form anywhere on its surface. We also provide multiple approaches to easily achieve localization, primarily based on controlling the electric field strength locally on the membrane, since the fabrication time is exponentially related to it (see Figure 3 and Figure S7). Our data suggest that a local change in thickness of 20-nm on the membrane can lead to a 10^4 change in fabrication time. One can readily envision how such an enormous difference in timescale can be leveraged to localize the pore formation on the membrane.

We demonstrate here such a strategy to localize the pore by patterning the surface of the membrane. While localization is shown at the micron scale, it is straightforward to envision how they could be scaled down to the sub-100-nm, and serves as a proof of concept.

As described in Section 4, we used specially designed chips to help in the acquisition of TEM images. The design of these chips was initially intended to reduce the chip parasitic capacitance and minimize high-frequency noise, which otherwise limit the bandwidth of nanopore recordings. We made use of the patterning of silicon nitride (SiN) membrane with a silicon dioxide (SiO_2) layer to localize the pore to a circular area ranging in diameter from 4 to 10-µm, on the otherwise square SiN membrane with sides ranging from 50 to 100-µm. Figure S12 shows a schematic representation of the patterned membrane, which is used to localize the pore formation.

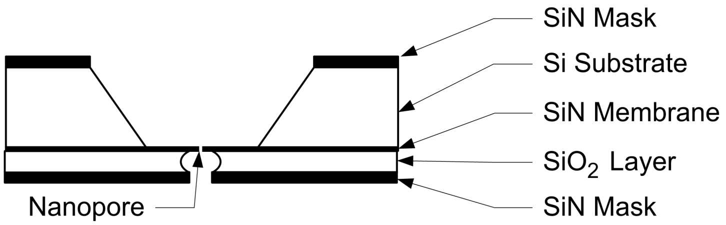

Figure S12: Schematic of the membranes provided by Stratos Genomics (not to scale). The silicon nitride (SiN) membrane is grown by LPCVD and is 38-nm ±4nm thick. The silicon dioxide (SiO_2) layer is grown by PECVD and is ~3-µm thick. The SiN mask is grown by PECVD and is ~100-nm thick. Opening in the SiN mask and SiO_2 layer range from 4 to 10-µm. [image used with permission of Stratos Genomics].

Surface patterning of the membrane allowed us in this case to localize the electric field lines to the portion of the SiN membrane not covered by SiO_2, and exposed to electrolyte solutions on both sides (see Figure S5 found in the supplementary information section 4).
